# The characterization of *OfRGA* in regulation of flower size through tuning cell expansion genes

**DOI:** 10.3389/fpls.2024.1502347

**Published:** 2024-12-20

**Authors:** Qianqian Wan, Mei Lu, Gege Jiang, Jiexin Shao, Tao Chen, Liyuan Yang, Irshad Ahmad Khan, Jinping Deng, Shiwei Zhong, Yiguang Wang, Zheng Xiao, Qiu Fang, Hongbo Zhao

**Affiliations:** Zhejiang Provincial Key Laboratory of Germplasm Innovation and Utilization for Garden Plants, School of Landscape and Architecture, Zhejiang A&F University, Hangzhou, Zhejiang, China

**Keywords:** *Osmanthus fragrans*, *OfRGA*, floral organ development, floral size, cell expansion

## Abstract

Flower appearance stands as a key characteristic of flowering plants and is closely linked to their ornamental value. Phytohormone Gibberellin (GA), essential for plant growth and development are widely reported for expansion in flower. DELLA proteins are known to negatively regulate GA signaling and influences plant growth and development through the regulation of cell expansion. However, the specific biological function of DELLA proteins in the woody plant *Osmanthus fragrans* remains unclear. In this study, *O. fragrans* ‘Sijigui’ was utilized as the experimental material, and *OfRGA* was isolated using the PCR method. *OfRGA* is expressed in various tissues and is localized in the nucleus. A negative association was observed between *OfRGA* expression and petal size across four different *Osmanthus fragrans* cultivars. Transformation experiments in tobacco revealed that transgenic plants overexpressing *OfRGA* exhibited increased plant height, greater node spacing, shorter leaf length, and wider leaves during the vegetative phase. Notably, the flower organs of transgenic tobacco plants displayed noticeable alterations, including reduced petal size, shorter corolla tubes, pedicels, male and female stamens, and lighter petal color. Furthermore, a decrease in the length and area of petal and corolla tube cells was observed as well. DEGs were found in RNA-seq studies of *OfRGA* transgenic plants. Subsequent investigation revealed a considerable quantity of down-regulated genes were associated with cell wall synthesis genes and expansion genes, such as *CesA1*, *XEH*, and *EXPB1*, as well as genes related to anthocyanin biosynthesis. Overall, our findings suggest that *OfRGA* undermines tobacco petal size by influencing cell expansion. The present study offers a fundamental comprehension of the role of DELLA protein in the organ development in *Osmanthus fragrans.*

## 1 Introduction

Floral organ development has profound significance in the plant life cycle, which is directly related to a plant’s ability to reproduce and the ecological adaptability of plants (Chahtane et al., 2023; Mā, 2005). This process is both finely regulated by internal physiological mechanisms and also greatly influenced by the external environment (Coen and Meyerowitz, 1991; Yue et al., 2023). The phytohormone gibberellin regulates the development of floral organs, according to rencent studies (Li et al., 2017, 2021).

Tetracyclic diterpenoids known as gibberellins are crucial for various critical processes in plant development, including fruit ripening, leaf expansion, floral organ development, stem elongation, seed germination, and flowering induction (Fleet and Sun, 2005). Previous research has identified that in mutants of *Arabidopsis thaliana* with deficiencies in GA synthesis, such as *ga1-1, ga1-3*, and *ga1-6*, the petals and stamens become damaged, and anthers become stunted in the absence of mature pollen, which causes male sterility in severe cases. However, exogenous GA can return the floral developmental phenotypes of *ga1-3* mutants to their normal state (Gotô and Pharis, 1999; Koornneef and van der Veen, 1980; Wilson et al., 1992). Likewise, in tomato, GA-deficient mutant *ga-2* has impaired flower development due to the lack of corolla and stamen elongation and the style deformation or elongation, leading to plant sterility. But the completion of bud development and fruit set was achieved by applying GA1 to developing inflorescences (Joan and Jan, 1988). The above results show that GA can control the flower development of plants. DELLA proteins are numbers of the plant-specific GRAS family and identified as negative regulators of GA. They have an inhibitory influence on plant development and growth. DELLA proteins are able to bind to GID1 to form a GA-GID1-DELLA trimer, which is labeled by the SCF polymers and subsequently eliminated by the ubiquitin 26S proteasome. *Arabidopsis thaliana* contains five DELLA proteins, identified as gibberellin insensitive (*GAI*), repressors of GA1-3 (*RGA*), *RGA*-Like1 (*RGL1)*, *RGL2*, and *RGL3* (Tyler et al., 2004). Both overlapping and specific functions are present in these five proteins. Among them, the growth of stamens, petals, and anthers in *Arabidopsis* is co-regulated by *RGA*, *RGL1*, and *RGL2* (Cheng et al., 2004 *)*. Further studies revealed that GA degrades DELLA primarily through the GA-GID1-DELLA pathway, thereby relieving the inhibitory effect on plant growth and development. In *Arabidopsis thaliana*, the inhibition of DELLA proteins *RGL1*, *RGL2*, and *RGA* by GA leads to the expression up-regulation of the floral homologous genes *AP3*, *PI*, and *AG*, which promote the normal development of floral organs (Cheng et al., 2004; Yu et al., 2004). *CsGAIP*, a homologue of DELLA protein in cucumber, inhibits the production of stamens in male cucumber flowers by downregulating the transcription of class B MADS-box genes *PI* and *AP3* (Zhang et al., 2014). Furthermore, the GA-DELLA-OsMS188 module controls rice anther development and integrates gibberellin hormone signaling into the anther genetic program (Jin et al., 2021).

Early studies have also found that biologically active gibberellins (GA) regulate plant floral organ development by modulating cell proliferation and expansion. In *Gerbera hybrida*, GA promotes petal elongation by enhancing cell expansion (Zhang et al., 2012). The phloem and xylem tissues in the peduncle of the “Wonhwang” oriental pear also expanded rapidly under the stimulation of exogenous GA (Park and Park, 2017). DELLA proteins, as key factors in the GA pathway, also play a role in regulating plant cell proliferation and expansion. DELLAs inhibited leaf growth through a dual mechanism, first by changing the rate of cell division during the proliferative phase of leaf development and then by effecting the rate of cell expansion while in the expansion phase (Achard et al., 2009). For example, cell proliferation in the meristematic zone might be inhibited by MpDELLA, which could restrict the growth of the liverwort *Marchantia polymorpha* (Hernández-García et al., 2021). In roses, *RhGAI1*, a target of *RhEIN3*-3, being silenced in rose petals has promoted cell expansion, which has resulted in enlarged petal size (Luo et al., 2013). In addition, DELLA proteins are not only solely responsible for regulating flower organ development but also have a certain effect on flower color. Research has found that TAP-GAId17 overexpression led to a significant rise in anthocyanins in transgenic *Arabidopsis* (Zhang et al., 2017). *MdRGL2a* can promote anthocyanin accumulation under the protection of the protein kinase MdCIPK20 in apple (An et al., 2023). The findings suggest that the DELLAs serve as positive regulators in the anthocyanin accumulation. This was investigated further and revealed that *RGA* interacts with *JAZ* and *MYBL2* in the MYB/bHLH/WD40 complex to release the bHLH/MYB subunit, which in turn forms an active MBW complex and hence anthocyanin biosynthesis (Xie et al., 2016). Currently, there are few studies on the regulatory mechanisms of DELLA proteins on the development of floral organs and flower color, which need to be further explored.

In ‘Sijigui’, we identified a gene encoding a DELLA protein (*OfRGA*), cloned the *OfRGA* gene by RT-PCR, and examined the patterns of *OfRGA* expression in various tissues and in various ‘Sijigui’ cultivars. We further heterologously transformed *OfRGA* into tobacco and investigated the gene function of *OfRGA*. The cell size of transgenic petals was observed by temporary sectioning. To gain a more profound understanding of the regulation mechanism of *OfRGA* on the development of floral organs, transcriptome sequencing was carried out. These investigations advance our knowledge of the molecular pathways through which *OfRGA* controls the development of floral organs.

## 2 Materials and methods

### 2.1 Plant materials and growing conditions

Four different cultivars of *Osmanthus fragrans* (‘Tianxiang Taige’, ‘Rixiang Gui’, ‘Sijigui’, and ‘Tiannu Sanhua’) plants were produced in a natural environment and provided through the resource garden at Zhejiang A&F University (Wang et al., 2022). The roots, stems, leaves, flower buds, leaf buds, petioles, stem segments, and other tissues of ‘Sijigui’ were gathered and kept at -80°C for subsequent experiments. The Zhejiang Provincial Key Laboratory of Garden Plant Germplasm Innovation and Utilization preserves tobacco of the wild type. Plants of the transgenic varieties and the wild type were cultivated at 23°C in a greenhouse with a 12 hours of light followed by 12 hours of darkness.

### 2.2 Gene cloning and bioinformatics analysis

There is a DELLA protein in *Osmanthus fragrans*, which is called *OfRGA*. The ORF sequence of *OfRGA* was obtained from transcriptome data. Gene-specific primers (listed in **Supplementary Table S1**) were used to amplify *OfRGA*. First-strand cDNA synthesized from *O. fragrans* ‘Sijigui’ was used as the PCR template. The amplification process consisted of an initial denaturation at 95°C for 3 minutes, followed by 32 cycles of denaturation at 95°C for 15 seconds, annealing at 60°C for 15 seconds, and extension at 72°C for 2 minutes. A final extension step at 72°C for 5 minutes completed the process.

The PCR product that had been gel-purified was cloned into the pMD18-T vector (TaKaRa, Dalian, China) and validated by forwarding it to the company for sequencing (Zhejiang Youkang Biotechnology Co. Ltd., Hangzhou, China).

Gene sequence homology was further analyzed using BLAST in the NCBI GenBank database (https://blast.ncbi.nlm.nih.gov). The phylogenetic tree was made with the MEGA 7.0 software. With the DNAMAN program, multiple sequence alignments and analyses were carried out (https://www.lynnon.com/dnaman.html) (Kumar et al., 2018).

### 2.3 Gene expression analysis by quantitative real-time PCR

The roots, flower buds, stems, leaf buds, leaves, petioles, and shoot apex of *O. fragrans* ‘Sijigui’ were collected as previously described. Total RNA was individually isolated from each tissue through the RNAprep Pure Plant Kit (Vazyme, Nanjing, China). Next, HiScript^®^ Reverse Transcriptase was utilized to generate the cDNA of the first strand (Vazyme, Nanjing, China) in accordance with the instructions provided by the manufacturer. **Supplementary Table S1** lists the specific primers that were created. RT-qPCR was carried out with a 10 uL PCR mixture that included 5 μL of SYBR Premix Ex Taq, 2 μL of cDNA, 0.4 μL each of PCR forward primer and reverse primer, and 2.2 uL of double-distilled (ddH2O). Then the internal reference gene and the target gene were added independently to a 96-well plate with three copies of each sample. The relative gene expression was computed using the 2-Ct method (Livak and Schmittgen, 2001).

### 2.4 Subcellular localization

The ORF of *OfRGA* lacking the stop codon was ligated into the pORER4-35S-GFP vector. And using the freeze-thaw method, the control, nuclear marker, and OfRGA-GFP expression plasmids were transferred into the *Agrobacterium rhizogenes* strain GV3101, where Agrobacterium expressing the plasmid and the control plasmid were mixed with Agrobacterium with the nuclear marker (35S::D53-RFP), respectively, and co-injected into the back of the tobacco leaves. The GFP fluorescence of tobacco leaves was detected after infiltration for 2 days to identify the protein’s position in the cells using a confocal microscope (Olympus Corporation, Tokyo, Japan) (Liu et al., 2023).

### 2.5 Tobacco transformation

Similarly, the ORF of *OfRGA* had been inserted into the vector pORER4 behind the 35S promoter, and the resulting construct 35S:*OfRGA* was transferred into the *Agrobacterium tumefaciens* strain GV3101 and then transformed into tobacco by the leaf disk method (Sparkes et al., 2006). Leaves were cultured in MS medium for 3 days. They were then moved to MS Medium, which included the following: 250 mg/L of carbenicillin disodium (Carb) + 2.25 mg/L of 6-benzyladenine (6-BA) + 0.3 mg/L of 1-naphthylacetic acid (NAA) + 100 mg/L of kana (a pH of 5.8). After being separated from the callus, the regenerated shoots were placed on the rooting media (MS + 100 mg/L Kana + 250 mg/L Carb) (a pH of 5.8). After rooting, the seedlings were moved to soil pots for cultivation. In order to observe phenotypic traits and compile data, transgenic plants that have been identified and WT control plants were grown in identical environments.

### 2.6 Microscopy examination and cell measurements

To observe the upper epidermal cells of the petals, the petals were collected and preserved for 24 hours at 4°C using a formaldehyde-acetic acid-alcohol (FAA) fixative (5% acetic acid, 5% formaldehyde, and 70% alcohol), followed by an alcohol rinse. Petal cells were then photographed using a confocal microscope (Olympus Corporation, Tokyo, Japan). The upper portion of the tobacco corolla tube was excised and transversely sectioned into several thin slices using a clean double-sided blade. These slices were then observed under the previously described confocal microscope. The area of the AbsE cells was measured using the ImageJ software (Rueden et al., 2017).

### 2.7 RNA sequencing and differential expression gene analysis

Using cDNA created from RNA extracted from tobacco flower bracts, differentially expressed genes (DEGs) between WT and transgenic lines of *OfGRA* were found using an Illumina sequencing platform (Beijing Bemac Biotechnology Co., Ltd., Beijing, China). Next, the Osmanthus fragrans reference geneome was mapped to the clean reads, and DEGs were examined for GO and KEGG enrichment. On the NCBI sequence read archive (SRA), the clean reads were submitted. (http://www.ncbi.nlm.nih.gov/sra/), accession number: PRJNA1122233. (https://www.ncbi.nlm.nih.gov/sra/PRJNA1122233).

### 2.8 Statistical analysis

Statistical significance was assessed using *p < 0.05, **p < 0.01, and ***p < 0.001 after data were processed with SPSS statistics and Excel software. There were three biological duplicates for each experiment.

## 3 Results

### 3.1 Sequence alignment and phylogenetic analysis

**Figure 1:**
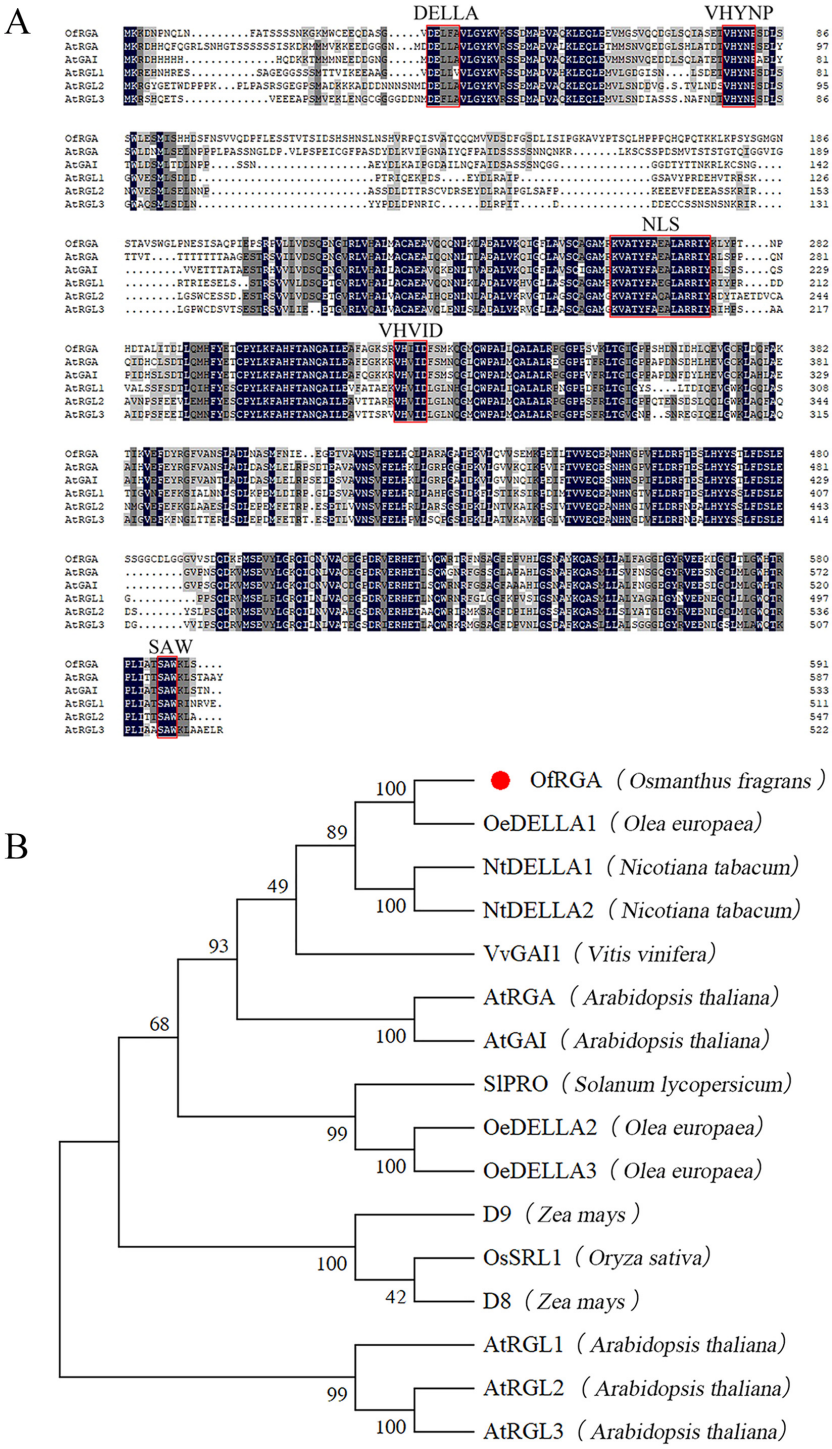
Sequence analysis of OfRGA. **(A)** Sequence alignment comparing *AtDELLAs* to *OfRGA*. DNAMAN was used for the alignment of amino acid sequences. Red lines surround the conserved domains of the DELLA protein. **(B)** The evolutionary connection between homologous genes and OfRGA. The symbol for OfRGA is a red circle. The following are the protein sequences: OfRGA (*Osmanthus fragrans*), OeDELLA1 (*Olea europaea* XP_022855476.1), OeDELLA2 (*Olea europaea* XP_022844159.1), OeDELLA3 (O*lea europaea* XP_022884592.1), NtDELLA1 (*Nicotiana tabacum* XP_016452673.1), NtDELLA2 (*Nicotiana tabacum* XP_016441385.1), VvGAI1 (*Vitis vinifera* NP_001384785.1), AtRGA (*Arabidopsis thaliala* NP_178266.1), AtGAI (*Arabidopsis thaliala* NP_172945.1), AtRGL1 (*Arabidopsis thaliala* NP_176809.1), AtRGL2 (*Arabidopsis thaliala* NP_186995.1), AtRGL3 (*Arabidopsis thaliala* NP_197251.1), OsSRL1 (*Oryza sativa* NP_001405437.1), ZmD8 (*Zea mays* NP_001354393.1), ZmD9 (*Zea mays* NP_001296780.1), SlPRO (*Solaum lycopersicum* NP_001234365.1).

The ORF of the *OfRGA* gene is 1776 bp in length and encodes 592 amino acids. Multiple sequence comparisons using DNAMAN software showed that all DELLA protein sequences have DELLA and TVHYNP motifs at the n-terminus and GRAS structural domains at the C-terminus, suggesting high consistency among DELLA proteins (**Figure 1A**). To further explore the evolutionary connection between *OfRGA* and other DELLA protein members, we compared 16 protein sequences from eight representative plant species and constructed a phylogenetic tree. According to the phylogenetic tree, *OfRGA* and the evolutionary history of *OeDELLA1* in *Olea europaea* are tightly linked (**Figure 1B**).

### 3.2 Expression and subcellular localization of *OfRGA*

**Figure 2:**
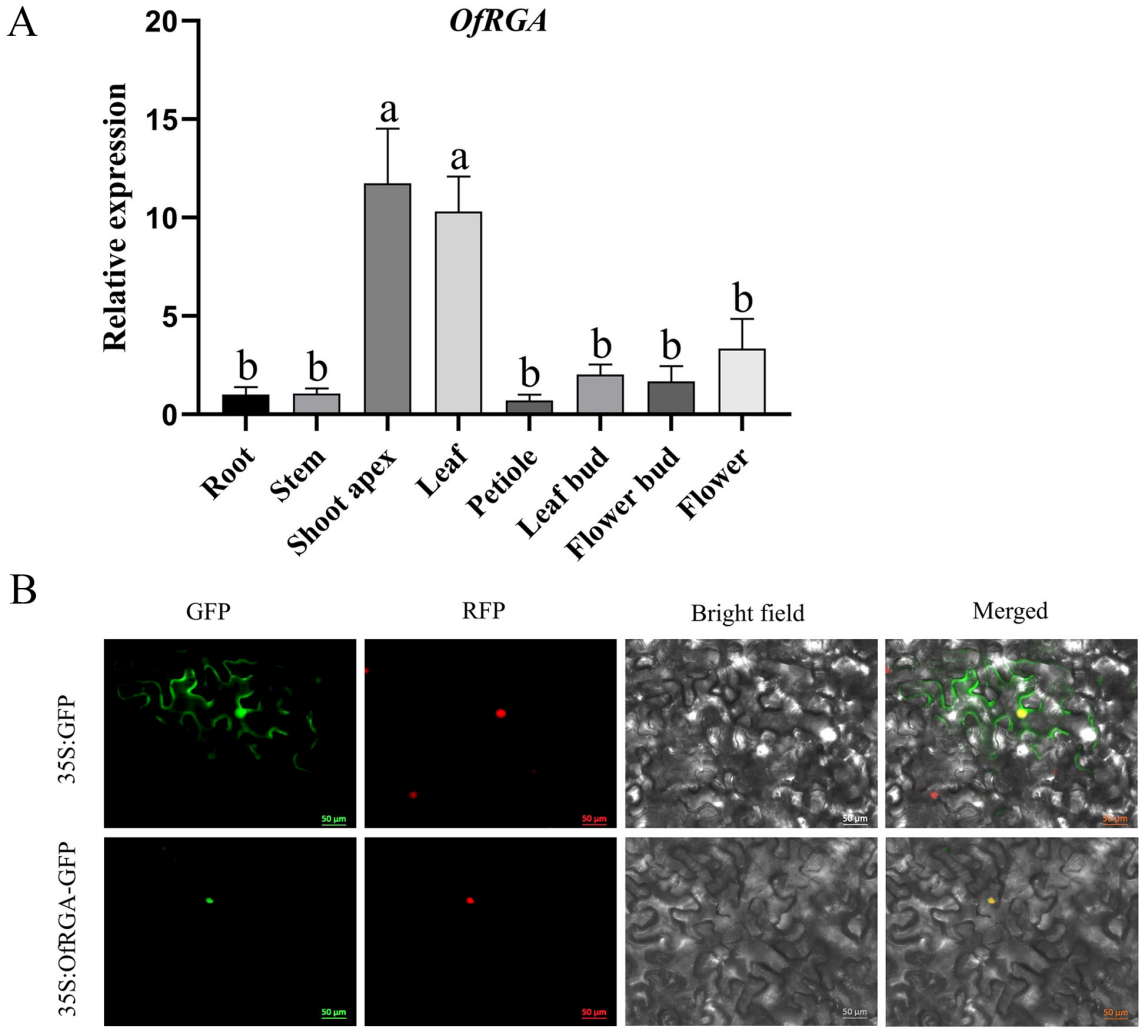
Expression and Subcellular Localization of *OfRGA*. **(A)** Profile of expression for *OfRGA* in *Osmanthus fragrans.* Quantitative real-time PCR examination of *OfRGA* in the leaf bud, petiole, stem, shoot apex, flower bud, root, and leaf of *Osmanthus fragrans*. Data are the means ± SD, n = 3, P < 0.05. **(B)** Location of *OfRGA* subcellularly in tobacco leaves. The nuclear marker signal is red, whereas the green fluorescent protein is green. Scale bars = 50 μm. a,b means α=0.05.

In order to further elucidate the role of *OfRGA*, qRT-PCR is used to detect the expression of *OfRGA* in the stems, roots, flower buds, petioles, leaves, leaf buds, and shoot apex of *O. fragrans* ‘Sijigui’ (**Figure 2A**). The results indicate that *OfRGA* has the maximum degree of expression in the shoot apex. According to subcellular localization experiments, the control GFP signal was dispersed throughout the cell, whereas OfRGA-GFP showed strong fluorescence signals in the nucleus. The finding demonstrated that the nucleus was where the *OfRGA* fusion protein was located (**Figure 2B**).

### 3.3 Analysis of *OfRGA* expression in the petals of different varieties of ‘Sijigui’

**Figure 3:**
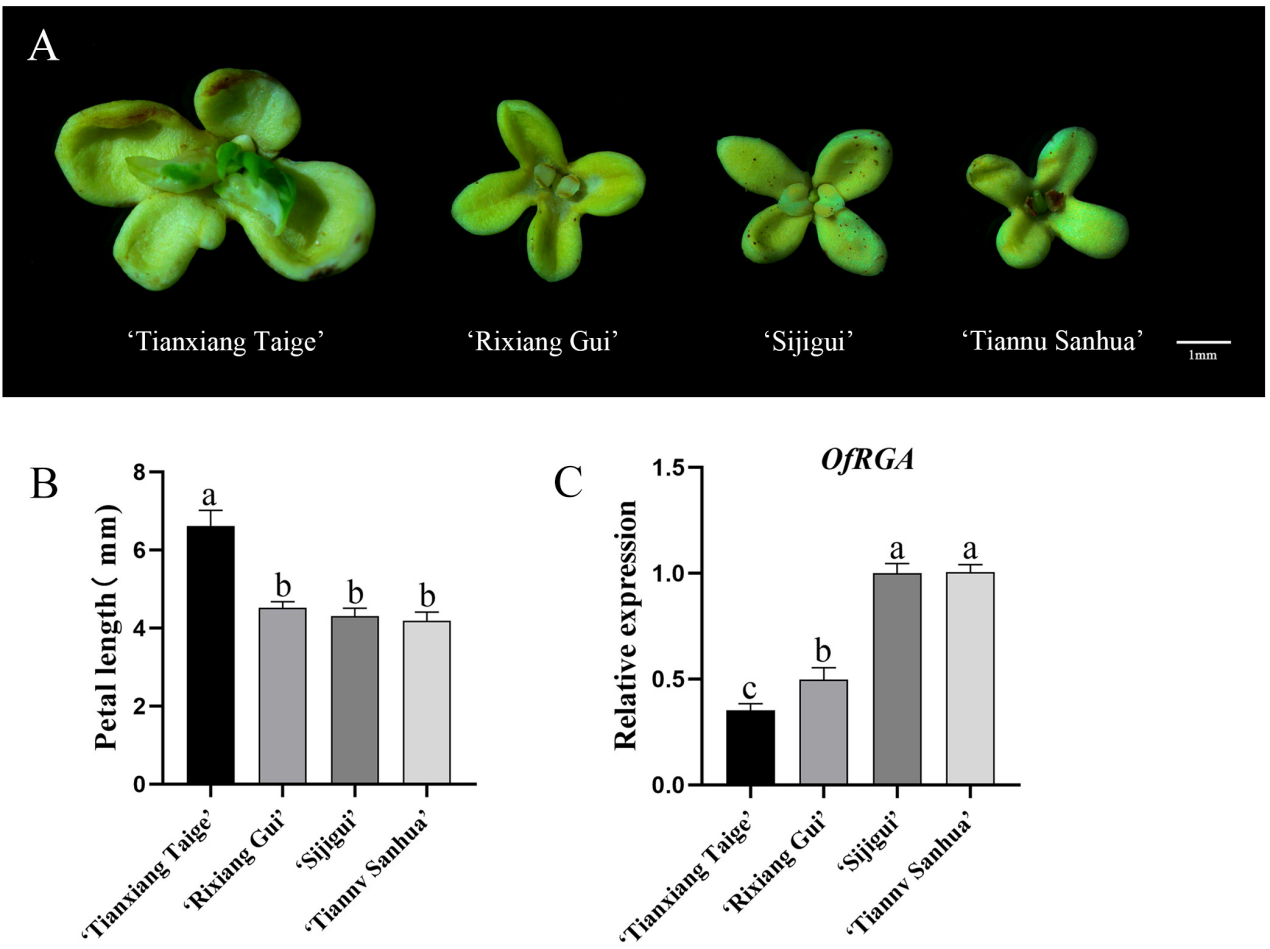
Analysis of *OfRGA* expression in the petals of various ‘Sijigui’ cultivars. **(A)** Flowers of different cultivars of ‘Sijigui’ (scale bar = 1 mm). **(B)** Measurement of petal length in different cultivars of ‘Sijgui’. **(C)** Gene expression of *OfRGA* in petals of different varieties. Data are the means ± SD, n = 3, P < 0.05. a,b,c means α=0.05.

Under the same growing environment, we observed a significant difference in petal size among four different cultivars of ‘Sijigui’. In order to understand this difference, four varieties, ‘Tianxiang Taige’, ‘Rixiang Gui’, ‘Putong Sijigui’, and ‘Tiannu Sanhua’, were selected for observation and measurement (**Figures 3A, C**). The results showed that the ‘Tianxing Taige’ variety had the largest petals, followed by ‘Rixiang Gui’, while ‘Putong Sijigui’ and ‘Tiannu Sanhu’ had approximately the same petal size. In order to investigate whether petal size is associated with the *OfRGA* gene, we quantitatively analyzed the petals of four different varieties of ‘Sijigui’ (**Figure 3B**). The analysis showed varying levels of *OfRGA* gene expression among the petals of the four different varieties, with a negative correlation between *OfRGA* expression and petal size. These findings suggest that *OfRGA* may play a role in limiting cell proliferation, thereby contributing to reduced petal size.

### 3.4 *OfRGA* overexpression alters tobacco development

**Figure 4:**
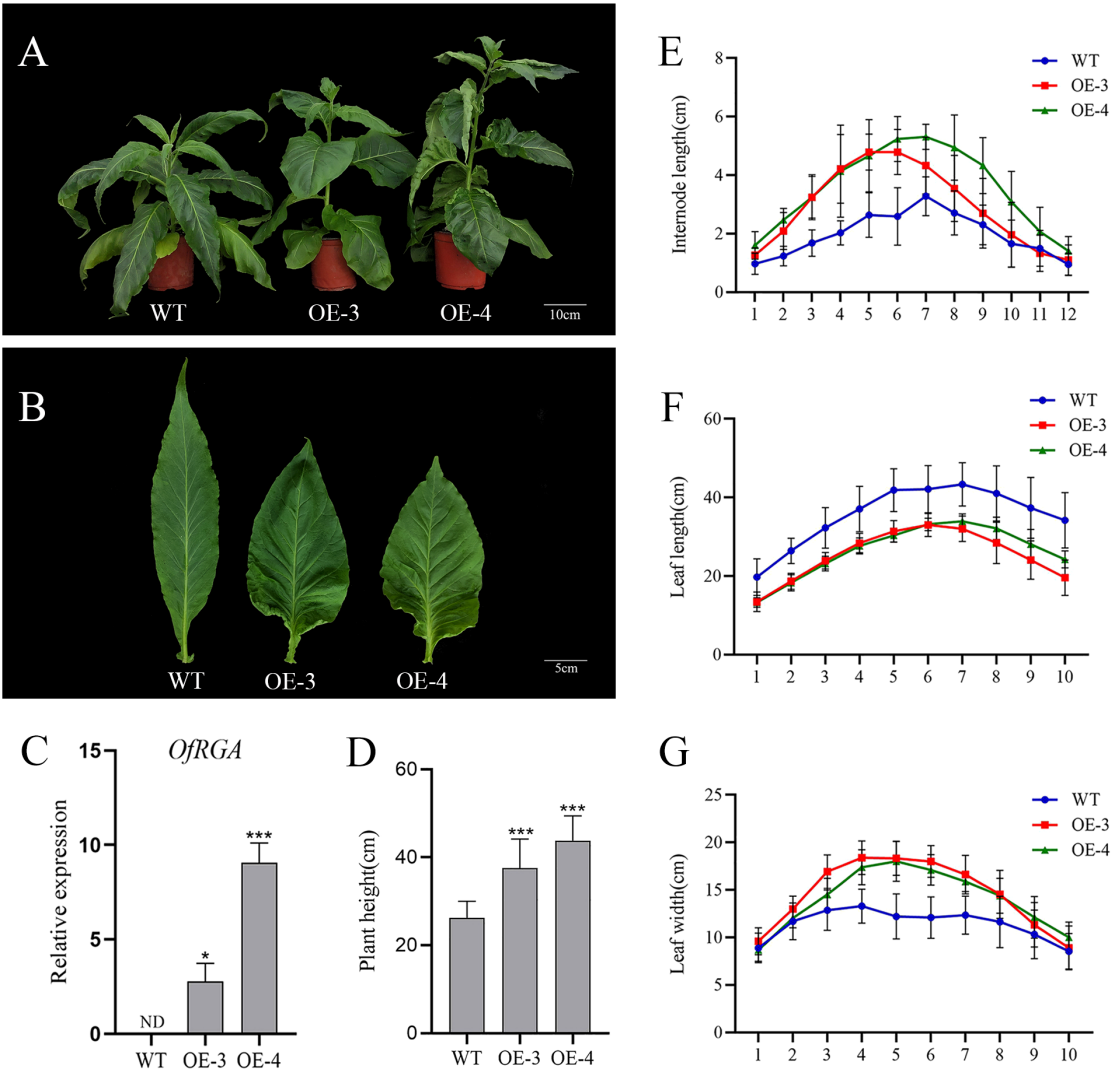
**(G)** Morphological phenotypes of tobacco overexpressing *OfRGA*. **(A)** Phenotypes of 73-day-old wild-type and transgenic plants. Scale bar = 10 cm. **(B)** Abnormal development of plant leaves. Scale bar = 5 cm. **(C)** Expression analysis of the *OfRGA* gene in transgenic lines. **(D–F)** Measure the plant height, internode length, leaf length, and leaf width of the wild type and transgenic lines. I1-I12, internode1-internode12; L1-L10, leaf1-leaf10. Error bars: ± SD. n = 10, *p < 0.05, ***P <0.001.

Agrobacterium-mediated transformation was used to create tobacco transgenic overexpression lines in order to further explore the gene function of *OfRGA*. From each construct, over ten distinct transgenic tobacco lines (35S::OfRGA) were produced, the vast majority of which had significantly altered phenotypes. For phenotyping purposes, we chose two transgenic lines that exhibit elevated levels of expression. The transgenic lines of *OfRGA* were all found to exhibit phenotypes of increased plant height, increased internode spacing, shorter leaf length, wider leaf width, and varying degrees of curling of the leaf margins when comparing the transgenic lines to the wild type (**Figures 4A–G**). These findings indicate that the overexpression of *OfRGA* promotes plant development and growth.

### 3.5 Development of floral organs is impacted by *OfRGA* overexpression

**Figure 5:**
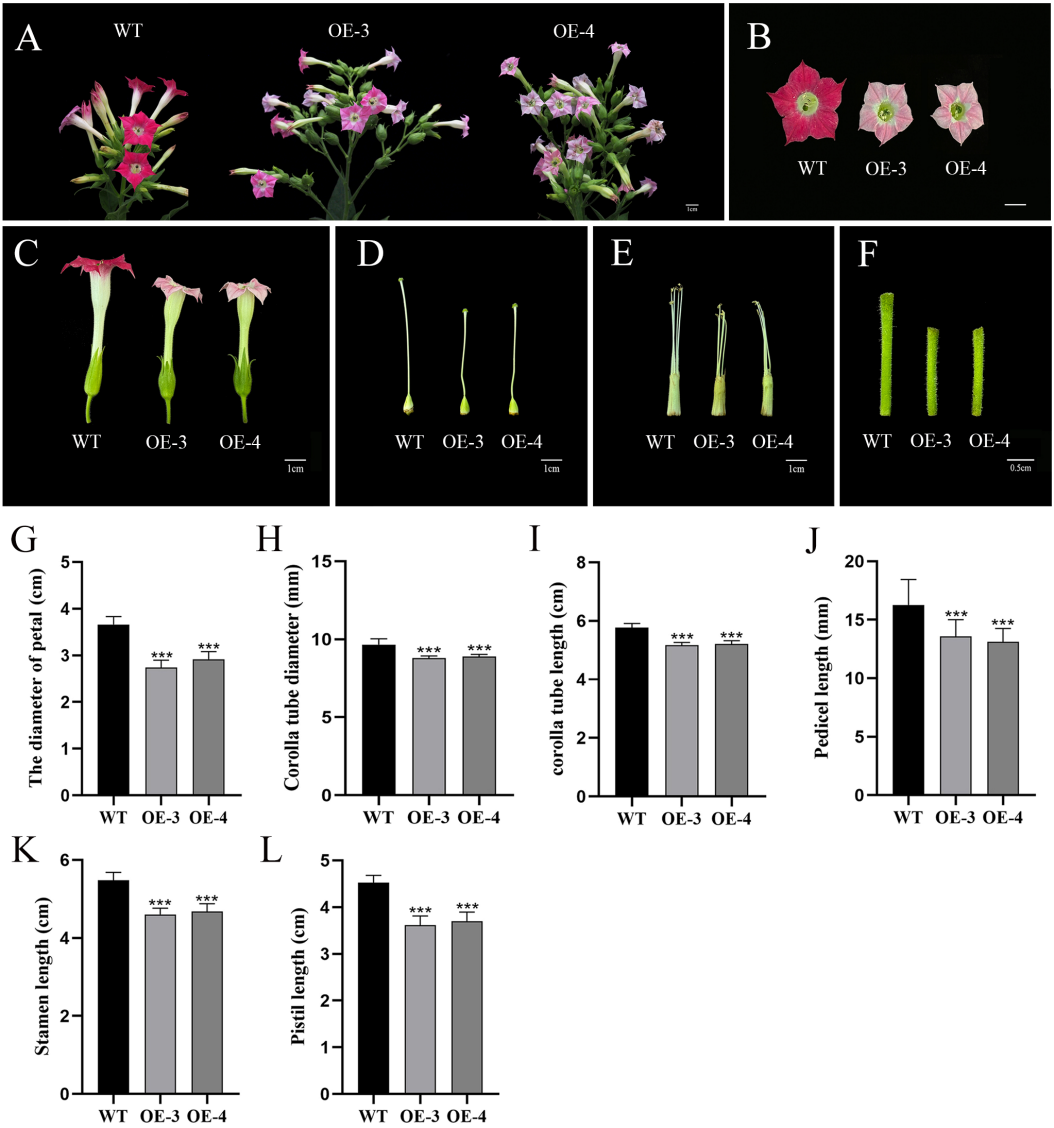
Effects of floral organ development modulated by overexpression of *OfRGA* in tobacco. **(A–F)** Phenotypic comparison of inflorescence, petals, corolla tube, pistil, stamen, and pedicel between wild-type and transgenic plants. **(G–L)** Comparison of petal diameter, corolla tube diameter and length, pedicel length, stamen length, and pistil length between plants that overexpress *OfRGA* and WT. Data were the means ± SD, n = 20, ***P <0.001.

After blooming, the phenotypes were further observed in the transgenic tobacco lines, and the outcomes demonstrated that the petals of the *OfRGA* transgenic line were significantly smaller and lighter in color than those of the wild type, further verification of the hypothesis that *OfRGA* inhibits cell expansion and reduces petal size (**Figures 5A, B**). In addition, to determine whether different floral organs’ growth and development were impacted, we also measured the width and length of the wild-type and transgenic corolla tubes, as well as the lengths of the stamens, pistils, and flower stalks (**Figures 5C–F**). We found that compared to the wild type, the petal diameter of OE-3 and OE-4 was reduced by 0.92 cm and 0.74 cm, the corolla tube diameter was reduced by 0.86 mm and 0.76 mm, the length of the corolla tube was reduced by 0.68 cm and 0.64 cm, the length of the pedicel was reduced by 2.7 mm and 3.17 mm, the length of the pistil was reduced by 0.91 cm and 0.82 cm, and the length of the stamen was reduced by 0.88 cm and 0.80 cm. The results indicate that the corolla tube of the transgenic strain became shorter and narrower, with reduced lengths of stamens, pistils, and pedicels compared to the wild type (**Figures 5G–L**). These findings confirm that the OE-*OfRGA* transgenic line exhibited significant reductions in the size and dimensions of these floral structures. In summary, the overexpression of *OfRGA* led to the shortening of floral organs.

**Figure 6:**
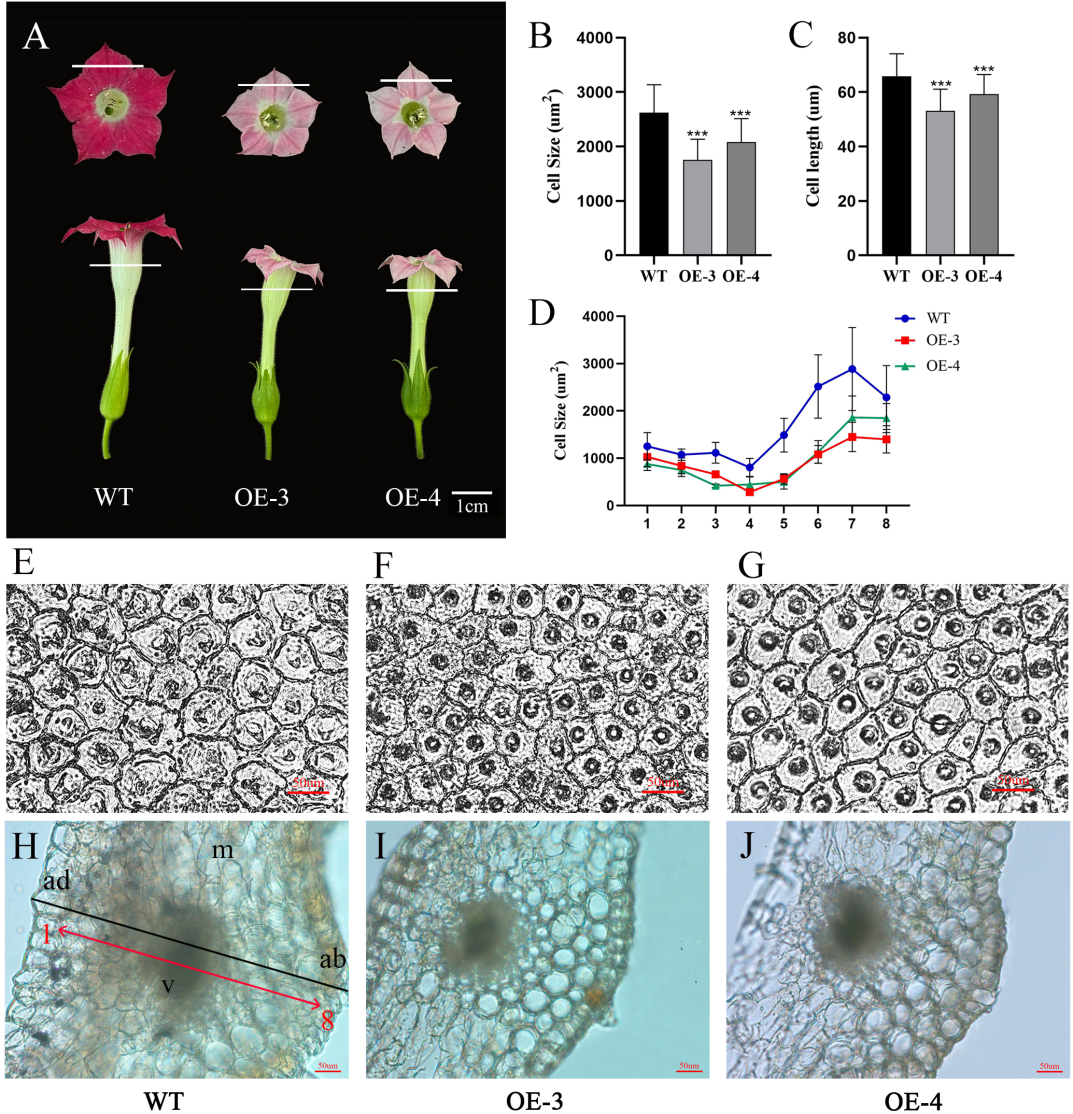
Overexpression of *OfRGA* can be attributed to a reduction in cell area and length, leading to a smaller petal. **(A)** Phenotypic petal and corolla tube. **(B, C)** Comparison of petal cell size and length in WT and overexpressing *OfRGA* plants. **(D)** Comparison of corolla tube cell size between WT and *OfRGA* overexpressing plants. **(E–G)** Using an optical microscope, the center portions of the petals of both WT and transgenic plants’ abaxial epidermal cells were studied. A 50 μm scale bar is used. **(H–J)** Cross-sections of the upper part of the corolla tube from WT and *OfRGA* overexpressing plants. ad, adaxial epidermal cell; ab, abaxial epidermis cell; m, mesophyll; v, vascular bundle. Bar = 50 µm. ***P <0.001.

To look into why the corolla tube is shorter and the petals are smaller, we conducted measurements on the cell area of both wild-type (WT) and transgenic petals, as well as corolla tubes (**Figures 6A, E–J**). We found that petal cell area decreased by 33.04% and 20.60%, as well as cell length decreased by 19.45% and 10.13% in OE-3 and OE-4, compared with the wild type (**Figures 6B–D**). It suggests that *OfRGA* may inhibit the growth of petals and corolla tubes by inhibiting cell expansion. Suggesting that *OfRGA* may inhibit petal and corolla tube growth by suppressing cell expansion.

### 3.6 RNA-seq revealed that *OfRGA* is targeted at multiple downstream genes engaged in the development of floral organs

**Figure 7:**
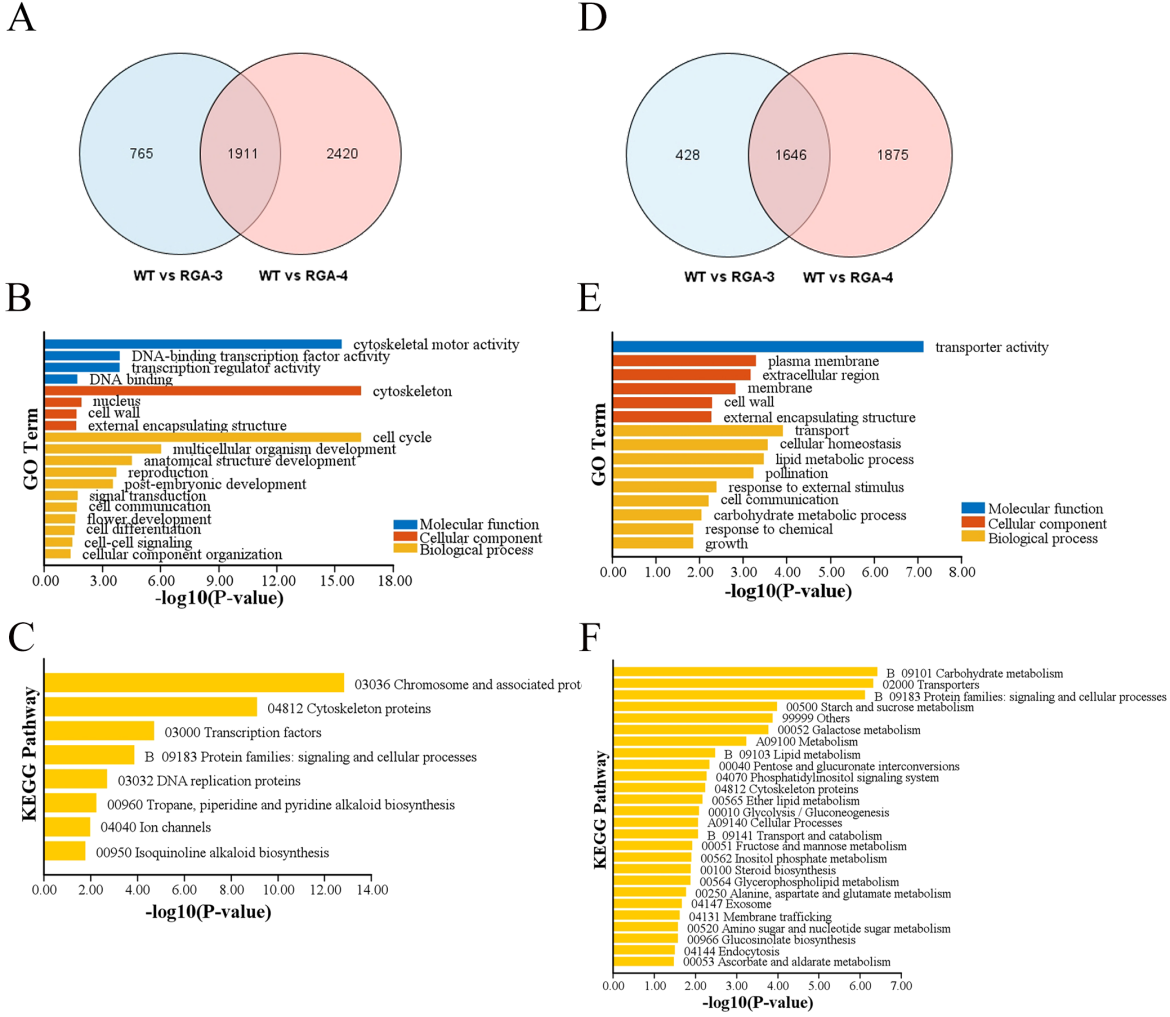
DEGs of transgenic tobacco with the *OfRGA* gene compared with WT. **(A)** Venn diagram showing how many up-regulated DEGs are shared and unique across the *OfRGA-3* and *OfRGA*-4 groups. **(B, C)** GO and KEGG analysis of shared up-regulated DEGs between *OfRGA*-3 and *OfRGA*-4. **(D)** Venn diagram showing how many down-regulated DEGs are shared and unique across the *OfRGA-3* and *OfRGA*-4 groups. **(E, F)** GO and KEGG analysis of shared down-regulated DEGs between *OfRGA*-3 and *OfRGA*-4.

To gain additional insight on the phenomenon through which *OfRGA* may inhibit the growth of petals and corolla tubes, as well as the causes of the lightening of flower color and the related genes through which *OfRGA* acts, differentially expressed genes (DEGs) were found using RNA-seq analysis. Transcriptomes from three biological replicates of WT and *OfRGA* overexpression transgenic plants were obtained for further bioinformatics analysis. Nine samples yielded 54 G of clean data, which was then used for additional bioinformatic analysis. 96.73% of the readings were successfully mapped to the Nicotiana genome. The DEG screen was conducted based on the criteria of a log2 fold change ≥ 1 and a Q value ≤ 0.05. 2,676 genes showed up-regulation and 2,074 genes showed down-regulation in WT compared to the OE-3 group, according to the analysis. Similarly, 4,331 genes showed up-regulation and 3,521 genes showed down-regulation in WT compared to the OE-4 group (**Figures 7A, D**). Taken together, genes that were up-regulated outnumbered those that were down-regulated. The GO analysis of WT versus OE-3 and OE-4 revealed that the up-regulated genes showed a high enrichment in molecular functions such as ‘DNA-binding transcription factor activity’ and ‘cytoskeletal motor activity’. In terms of cellular components, these genes were significantly enriched in the ‘nucleus’ and ‘cell wall’. The most significant enrichment was observed in the biological processes of ‘cell cycle’ and ‘multicellular organism development’ (**Figure 7B**). The molecular functions of the down-regulated genes were primarily abundant in ‘transporter activity’, the cellular components were primarily abundant in ‘plasma membrane’ and ‘extracellular region’, and the biological processes were mainly enriched in ‘transport’ and ‘cellular homeostasis’ (**Figure 7E**). According to KEGG enrichment analysis, the majority of the up-regulated genes were enriched in ‘Chromosome and associated proteins’, ‘Cytoskeleton proteins’, ‘Transcription factors’, and ‘Protein families: signaling and cellular processes’ (**Figure 7C**). while the down-regulated genes were gratetly enriched in these pathways, such as ‘Starch and Sucrose Metabolism’, ‘Carbohydrate Metabolism’, ‘Transporters’, and ‘Protein Families: Signaling and Cellular Processes’ (**Figure 7F**). These results indicate that genes and DNA-binding transcription factors involved in regulating the cell wall and cell cycle could be crucial in the organ development and cell expansion of transgenic tobacco flowers.

**Figure 8:**
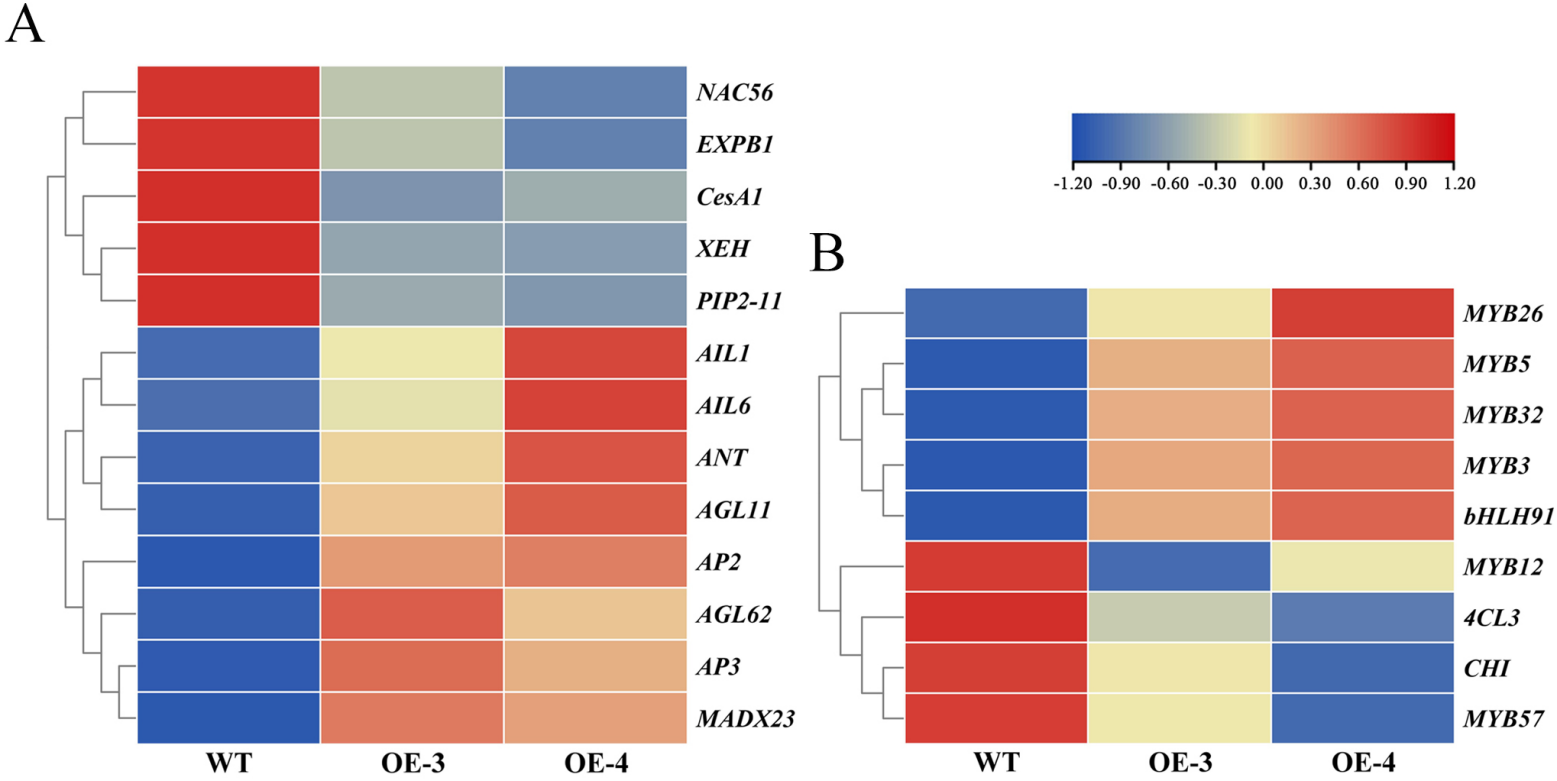
Expression heatmap of the DEGs. **(A)** Heatmap of floral organ development. **(B)** Heatmap of anthocyanin synthesis-related genes.

A total of 13 genes associated with the development of floral organs were identified from differentially expressed genes (DEGs), including the MADS-box gene family, AP2/ERF gene family, xyloglucan endotransglucosylase/hydrolase (*XTH*), Cellulose Synthase A1 (*CESA1*), Expansin B1 (*EXPB1*), *NAC56*, and Plasma Membrane Intrinsic Protein2-7 (*PIP2*-7). Among these, the MADS-box gene family and AP2/ERF gene family-related genes were significantly upregulated, while *XTH*, *CesA1*, *EXPB1*, *NAC56*, *PIP2-7*, and other genes participating in cell wall synthesis and cell expansion were significantly down-regulated (**Figure 8A**). These findings indicate that genes related to cell expansion are essential to the regulation of floral organ development. Furthermore, several DEGs associated with anthocyanin synthesis and regulation were identified. Genes involved in anthocyanin synthesis, such as Chalcone Isomerase (*CHI*), 4-Coumarate: Coenzyme A Ligase 3 (*4CL3*), MYB transcription factors *MYB12*, and *MYB57* were significantly down-regulated, whereas genes related to anthocyanin regulation, like MYB transcription factors *MYB26*, *MYB5*, *MYB32*, *MYB3*, and bHLH transcription factor *bHLH9*, were markedly up-regulated (**Figure 8B**). These results suggest that the color fading of transgenic plants may be due to the declined expression of anthocyanin biosynthesis-related genes and the upregulation of transcription factors such as *MYB* and *bHLH*. The exact results still need to be further verified through more experiments.

## 4 Discussion

One of the most attractive features of *Osmanthus fragrans* is its floral organs. The regulation of floral organ development is significantly influenced by phytohormones. DELLA protein, as a central element of the phytohormone regulatory network, is engaged in the communication and control of several hormones (Navarro et al., 2008). Here, we analyzed the DELLA protein *OfRGA* transcript levels in the petals of four different ‘Sijigui’ varieties, indicating that the DELLA protein *OfRGA* may play a role in limiting cell proliferation, thereby contributing to reduced petal size. In a previous study, DELLA proteins were found to take part in nearly all plant growth and development processes (Thomas et al., 2018). *AtRGL2* plays a role in regulating floral development in *Arabidopsis thaliana*. The expression of a dominant GA-resistant version of *RGL2*, resulted in a phenotype characterized by reduced stamen and petal size, as well as inhibited anther dehiscence (Gómez et al., 2019). In poplar, overexpressing *PmRGL2* from Japanese apricots delayed the beginning of bud dormancy and produced dwarfed plants (Lv et al., 2018). The tomato DELLA protein *SlPRO* participates in fruit development, flower development, and floral induction in tomatoes (Martí et al., 2007; Silva et al., 2018). However, information regarding the precise role of DELLA proteins in “Sijigui” is lacking.

With tobacco that has been heterologously transformed, the function of the *OfRGA* gene has been further investigated. The findings demonstrated that the *OfRGA* transgene displayed shorter and wider leaves, as well as increased plant height and node spacing when compared to the wild type (**Figure 4**). Early research has demonstrated that transgenic tobacco produces a dwarf phenotype when *AtGAI* and *DoDELLA* are overexpressed (Hynes et al., 2003; Zhou et al., 2022). And the same dwarfing phenotype was also observed in *Liriodendron hybrids* (Liu et al., 2020). The *OfRGA* transgenic plant height phenotypes differed from those reported in the existing species, but the protein sequences were found to be not significantly different by amino acid sequence comparison with those of the model plant Arabidopsis thaliana (**Figure 1A**). Therefore, it is speculated that it may be because of the different regulation of downstream structural genes by *OfRGA* during the growth and developmental stages of tobacco. This suggests a potential different regulation of downstream structural genes by *OfRGA* during the growth and developmental stages of tobacco. Furthermore, *OfRGA* was found to be involved in flower development in tobacco; the OE-*OfRGA* transgenic lines exhibited a significant reduction in the size and dimensions of these floral structures (**Figure 5**). This is similar with previous research findings on DELLA in other species. The DELLA homologue *CsGAIP* in cucumber suppresses stamen development by repressing homozygous heterozygous genes in class B flowers (Zhang et al., 2014). *Arabidopsis RGA*, *RGL1*, and *RGL2* all function to inhibit stamen, petal, and anther development in *Arabidopsis* (Cheng et al., 2004). Coordinated cell division and expansion during flower formation determine the size of floral organs (Mizukami and Fischer, 2000). Therefore, to further investigate the reasons for the smaller floral organs in overexpression of *OfRGA* transgenic tobacco, we performed freehand sections of petals as well as corolla tubes. The results showed a decrease in petal area as well as length in the transverse section of the upper portion of the corolla tube compared to WT (**Figure 6**). The above results show that the smaller size of transgenic tobacco floral organs may be due to a reduction in cell area and length. Further exploration of the function of the *OfRGA* gene requires *in vivo* verification through the acquisition of transgenic *Osmanthus* plants.

The entire set and quantity of transcripts present in a cell at a specific physiological state or developmental stage is known as the transcriptome, which is significant for understanding the level of gene expression, and exposing the molecular mechanism of biological gene expression regulation research (Qi et al., 2011). Using transcriptome technology, the primary mechanism of calcium-induced anthocyanin production in grapevine pericarp was discovered (Yu et al., 2020). Ninebark transcriptome analysis postulated that *BgSEP2b* is a center gene, interacting with multiple transcription factors and carpel-related genes, indicating that it might be an important gene for the development of ninebark carpels (Zhang et al., 2022). To further investigate the specific molecular mechanism by which *OfRGA* overexpression in tobacco leads to smaller floral organs, we examined transgenic tobacco using RNA-seq sequencing. And the transcriptome data demonstrated that genes associated with the expression of cell wall synthesis and proliferation, such as *CesA1*, *XEH*, and *EXPB*, were notably down-regulated in the transgenic strain when contrasted with the wild type (**Figure 8**). The size of plant organ is regulated by cell proliferation and expansion (Tsukaya, 2006). In the model plant *Arabidopsis thaliana*, *AtEXPA15* and *AtERF* regulate petal and fruit size by promoting cell division and thereby regulating petal and fruit size (Bernal-Gallardo et al., 2024; Lee et al., 2023). Furthermore, *OfXTH* genes might take part in the opening process of *Osmanthus* flowers by regulating cellular petal expansion (Yang et al., 2022). Earlier studies revealed that DELLA proteins control cell expansion and proliferation, which contribute to plant growth and development. *RhGAI1* overexpression inhibits petal cell expansion in *Arabidopsis thaliana*, and *RhGAI1* is engaged in the regulation of rose petal size by inhibiting the expression of *RhCesA2* (Luo et al., 2013). *BraRGL1* is a DELLA protein in Brassica rapa that represses the transcriptional activation of *BraXTH* by *BraSOC1* to inhibit the expansion of stalk (Wang et al., 2023). Together, these results imply that *OfRGA* may inhibit cell expansion by lowering the level of gene expression such as *CesA1*, *XEH*, and *EXPB*, resulting in smaller petals and corolla tubes of transgenic tobacco. However, it is still unclear whether the differences in petal size among four different varieties of ‘Sijigui’ are caused by *OfRGA* regulating the *EXPB*, *XEH*, and *CesA1* genes, and further experiments are needed to verify this.

Previous studies have found that the DELLA protein in *Arabidopsis* promotes anthocyanin biosynthesis by isolating the *JAZ* and *MYBL2* genes in the MBW complex or by interacting to enhance the transcriptional activity of PAP1/MYB75 (Xie et al., 2016; Zhang et al., 2017). Nevertheless, in this paper, the transgenic petals were significantly lighter in flower color. And further transcriptome analysis demonstrated that following O*fRGA* overexpression, the expression of the anthocyanin synthesis gene *CHI* was markedly down-regulated, whereas the anthocyanin-regulated transcription factors *MYB* and *bHLH* were significantly up-regulated (**Figure 8**). The *CHI* gene is an important structural gene in the anthocyanin synthesis process (Sun et al., 2019). Given the results above, it is speculated that introducing *OfRGA* through heterologous transformation could impact the expression of endogenous anthocyanin synthesis and regulatory genes in tobacco. Furthermore, studies have reported that *Osmanthus fragrans* contains anthocyanins (Shi et al., 2021). However, whether *OfRGA* mediates the synthesis pathway of anthocyanins in *Osmanthus fragrans* requires further research.

## 5 Conclusion

This research quantitatively examined the expression levels of *OfRGA* in the petals of four different ‘Sijigui’ varieties and observed the phenotypic changes in *OfRGA* transgenic tobacco, revealing the potential role of *OfRGA* in controlling the size of plant floral organs. Further results of slicing experiments and analysis of transgenic plants’ transcriptome data revealed that *OfRGA* might influence the size of plant petals by regulating genes related to cell expansion, such as *CesA1*, *XEH*, and *EXPB1*. These findings suggest that *OfRGA* might be crucial for the development of the organs in the *Osmanthus fragrans* flower and establish the framework for additional revelation of the molecular mechanism of *OfRGA* in *Osmanthus fragrans* flower organ development.

## Data Availability

The datasets presented in this study can be found in online repositories. The names of the repository/repositories and accession number(s) can be found below: https://www.ncbi.nlm.nih.gov/genbank/, PRJNA1122233.
