# Supplementary material for: The characterization of OfRGA in regulation of flower size through tuning cell expansion genes
Source: Front Plant Sci. 2024 Dec 20;15:1502347. doi: 10.3389/fpls.2024.1502347 (PMC11736142; doi:10.3389/fpls.2024.1502347)
Supplement: Supplementary file 1 [file Table1.docx]

**Table S1 primer sequences**

| Primer name | sequences（5^，^-3^，^） | Amplification purpose |
| --- | --- | --- |
| *OfRGA*-R | TGAAATTGGCCGAGGCTTTG | qRT-PCR |
| *OfRGA*-F | TAAGGCGGTGTCATGTGGAT | qRT-PCR |
| *OfRGA*-R4-F | TTCTTCACTGTTGATAGCTAGCATGAAGAAGGATAATCCGAATCAAC | Plant expression vectors |
| *OfRGA*-R4-R | AGGCCTTCTAGAAAAACTCGAGGCTGAGTTTCCAGGCCGAGGTA | Plant expression vectors |
| *OfACT*-F | CCCAAGGCAAACAGAGAAAAAAT | *O. fragrans* reference gene |
| *OfACT*-R | ACCCCATCACCAGAATCAAGAA | *O. fragrans* reference gene |
| *Nt26S-RNA*-F | GAAGAAGGTCCCAAGGGTTC | *Nicotiana* reference gene |
| *Nt26S-RNA*-R | TCTCCCTTTAACACCAACGG | *Nicotiana* reference gene |
